# Multimodal Hydrogel-Based Sensors for Sleep Respiratory Monitoring: Signal Sensing Mechanisms and Structural Design Strategies

**DOI:** 10.3390/s26185980

**Published:** 2026-09-21

**Authors:** Yuanfeng Sun, Liangchao Li, Yuan Shi, Jian Jiao, Taomei Li, Xiangdong Tang, Yihao Long, Liang He, Jingfei Xu, Lan Zhang

**Affiliations:** 1 Sleep Medicine Center, West China Hospital, Sichuan University, Chengdu 610041, China; sunny@wchscu.edu.cn (Y.S.); shiyuan@wchscu.cn (Y.S.); jiaojian@wchscu.edu.cn (J.J.); maytaomeili@hotmail.com (T.L.); tangxiangdong@scu.edu.cn (X.T.); 2 School of Mechanical Engineering, Sichuan University, Chengdu 610065, China; liliangchao1127@163.com (L.L.); longyihao17@stu.scu.edu.cn (Y.L.); heliang007@tsinghua.org.cn (L.H.); 3 Mental Health Center, West China Hospital, Sichuan University, Chengdu 610041, China; 4 National Center for Mental Disorders, West China Hospital, Sichuan University, Chengdu 610041, China; 5 Rehabilitation Medicine Center, West China Hospital, Sichuan University, Chengdu 610041, China; 6 Key Laboratory of Rehabilitation Medicine in Sichuan Province, West China Hospital, Sichuan University, Chengdu 610041, China

**Keywords:** hydrogel-based sensors, multimodal sensors, sleep respiratory

## Abstract

**Highlights:**

1. Multimodal hydrogel-based sensors combining respiratory motion, airflow, auxiliary physiological signals, and AI-assisted analysis provide comprehensive solutions for sleep respiratory monitoring.
2. Structural design strategies of hydrogel-based sensors are analyzed and summarized to elucidate their roles in signal integration, interference reduction, and long-term wearable monitoring.

**What are the main findings?**

- Hydrogel-based sensors can acquire diverse physiological signals associated with sleep-related breathing through mechanical, humidity, thermal, and electrophysiological sensing mechanisms.
- Structural design strategies, including single-layer, multilayer, biomimetic, and integrated structures, enable improved multimodal signal acquisition and reduced signal interference.

**What are the implications of the main findings?**

- Multimodal hydrogel-based sensors provide a promising platform for non-invasive and continuous monitoring of sleep-related respiratory states and associated physiological signals.
- Future development should focus on improving long-term stability, signal accuracy, multimodal decoupling, and clinical validation to facilitate practical sleep respiratory monitoring.

**Abstract:**

Sleep-related breathing disorders, particularly the obstructive sleep apnea syndrome, lead to an urgent demand for continuous, comfortable, and accurate monitoring technologies. However, conventional polysomnography is limited by complex procedures, poor wearing comfort, and restricted suitability for long-term monitoring. Owing to their skin-like mechanical performance, high biocompatibility, high ionic conductivity, and excellent tissue compatibility, hydrogels have emerged as promising materials for flexible bioelectronic devices. Recent multimodal hydrogel-based sensors enable simultaneous acquisition of mechanical, thermohygrometric, and electrophysiological signals, providing new opportunities for comprehensive sleep respiratory monitoring. This review systematically discusses the mechanisms for acquiring and analyzing multimodal physiological signals, including respiratory motion, respiratory airflow, auxiliary physiological signals, and AI-assisted sleep respiratory state recognition. Furthermore, structural design strategies, including single-layer, multilayer, biomimetic, and integrated architectures, are reviewed to clarify how structural configurations facilitate multimodal signal acquisition, signal interference suppression, and sensing performance optimization. This review provides valuable references for the future development of high-performance wearable multimodal hydrogel-based sensors toward sleep respiratory monitoring.

## 1. Introduction

Sleep-disordered breathing, particularly the obstructive sleep apnea syndrome (OSAS), has become a prevalent health concern with significant impacts on human health [1,2]. Recurrent episodes of apnea and hypopnea not only impair sleep quality but may also contribute to severe health complications, including cardiovascular diseases [3]. Therefore, the development of continuous, non-invasive, and long-term sleep-breathing monitoring technologies is of great importance for early disease screening and personalized health management [4]. Traditional polysomnography (PSG) is an important reference method for the clinical assessment of sleep-disordered breathing. It simultaneously records multiple physiological signals, including electroencephalography (EEG), electrooculography (EOG), electromyography (EMG), electrocardiography (ECG), respiratory airflow, chest and abdominal movements, and blood oxygen saturation, and evaluates the severity of sleep apnea based on the apnea–hypopnea index (AHI) [5,6]. However, conventional PSG typically requires multiple sensors, wired connections, and specialized equipment and personnel, which increases the complexity of the monitoring process and may cause discomfort to subjects. These limitations restrict its applications in home-based monitoring and long-term continuous sleep respiratory monitoring [7].

In recent years, the advances in flexible wearable sensors have introduced new opportunities for sleep health monitoring. Early studies primarily focused on single-mode sensing, however, individual physiological signals are often vulnerable to environmental disturbances and motion artefacts, which can compromise monitoring reliability [8,9]. Because sleep-related breathing disorders involve changes in multiple physiological processes, monitoring a single signal may provide very limited information about the underlying respiratory state. This has motivated the development of multimodal sensing systems that combine complementary physiological signals or sensing responses within a single wearable platform [10]. In this context, multimodal sensing encompasses the coordinated acquisition of two or more distinct types of physiological information, which may be obtained through different sensing mechanisms or from different physiological signals [11]. For sleep respiratory monitoring, these modalities include mechanical signals associated with thoracoabdominal movements, humidity or thermal signals associated with respiratory airflow, and auxiliary physiological signals such as pulse and electrophysiological signals. By combining complementary information from these modalities, multimodal systems have improved robustness of respiratory-state assessment and provide additional information for sleep-stage classification, apnea event identification, and overall health assessment [12,13].

Owing to their high water content, mechanical flexibility, biocompatibility, and tissue-like mechanical performance, hydrogels are emerging as highly promising candidates for the development of wearable bioelectronic devices [14]. Through the integration of conductive networks, functional components, and structural engineering strategies, hydrogel-based sensors have evolved from single-stimulus-responsive devices into multifunctional sensing platforms capable of simultaneously acquiring mechanical, thermohygrometric, and electrophysiological signals [15]. Nevertheless, existing reviews have mainly focused on wearable sensors for monitoring sleep-related breathing abnormalities or flexible and multimodal sensing platforms for respiratory monitoring, providing broad overviews of sensing technologies and their applications [7,11]. However, the specific role of hydrogels and structural engineering in enabling multimodal physiological signal acquisition for sleep respiratory monitoring has not been systematically discussed. In particular, the relationships between hydrogels’ structure and the detection of different physiological signals, as well as the roles of structural strategies in functional integration and signal interference suppression, remain insufficiently addressed.

Therefore, this review focuses on hydrogel-based multimodal sensors for sleep respiratory monitoring, with particular emphasis on physiological signal acquisition and analysis, and the structural strategies that have influences on their integration and characteristics. From the perspective of signal sensing, we systematically discuss the mechanisms by which hydrogel-based sensors acquire respiratory movements, respiratory airflow, and auxiliary physiological signals, including pulse and electrophysiological signals, as well as AI-assisted analysis for sleep respiratory state recognition. From the perspective of device architecture, we further examine how single-layer, multilayer, biomimetic, and integrated structures facilitate multimodal signal acquisition and reduce signal interference.

## 2. Mechanisms of Acquiring Physiological Signals Associated with Sleep-Related Breathing

### 2.1. Mechanical Response-Based Detection of Respiratory Movements

During sleep, periodic expansion and contraction of the thoracic and abdominal cavities reflect respiratory rhythm and ventilation status, serving as critical physiological indicators for sleep-related breathing monitoring [16,17]. Hydrogel-based respiratory sensors based on mechanical responses achieve signal transduction by converting deformation-induced changes within internal sensing networks into electrical outputs. These sensors primarily rely on mechanisms such as piezoresistive, capacitive, piezoelectric, and triboelectric effects [16,18,19,20]. When the chest and abdomen undergo periodic movements during respiration, corresponding variations in electrical signals, including resistance, capacitance, and current, are generated within hydrogel-based sensors [21,22].

According to the piezoresistive mechanism, mechanical deformation can be converted into electrical responses by modulating the internal conductive network or ionic transport pathways of the hydrogel, resulting in the changes in electrical resistance [23,24]. For ionically conductive hydrogels, mechanical deformation primarily influences their ion migration pathways and overall conductivities [25,26]. In contrast, for hydrogels incorporating metals, carbon-based materials, or MXene, the resistive responses are mainly achieved by regulating the contact state among conductive components and the electronic transport networks [27,28]. Owing to their simple device architecture, high sensitivity, and straightforward signal acquisition, piezoresistive sensors have been widely employed in wearable healthcare devices, motion monitoring, electronic skin, and human–machine interfaces [29]. Nevertheless, hydrogel-based piezoresistive sensors may suffer from signal drift and reduced long-term repeatability under repeated mechanical loading, which remain important challenges for their practical applications [23].

By capacitive sensing, the mechanical stimuli can be detected by monitoring variations in electrode distance, effective overlapping area, or dielectric behavior induced by deformation [30,31]. Capacitive sensors exhibit several advantages, including low power consumption, rapid response, and excellent signal stability. However, their sensing performance is highly dependent on the design of the dielectric layer and device microstructure, while parasitic capacitance, nonlinear responses, and environmental interference may limit their practical applications [19].

The piezoelectric sensing mechanism is based on the generation of electrical polarization under mechanical stress, where displacement of internal positive and negative charge centers produces a potential difference across the material, resulting in direct voltage or current output. Consequently, piezoelectric sensors can be regarded as self-powered sensing devices [32]. However, they generally exhibit limited capability for continuous static-pressure detection because electrical signals are generated primarily during dynamic deformation [20].

Triboelectric sensing relies primarily on electrostatic induction and contact electrification. When two materials with different electron affinities have repeated contact and separation, charge transport occurs at their interfaces, generating an electrostatic field that drives electron flow through an external circuit. This process produces alternating voltage or current signals and enables the conversion of mechanical energy into electrical energy [33,34]. Triboelectric sensors are another important class of self-powered sensors for detecting external mechanical stimuli. Compared with piezoelectric sensors, triboelectric sensors can generate substantially higher output voltages. However, their performance in static pressure sensing is often limited by signal attenuation, rapid charge dissipation, and long-term output instability [7,35].

**Figure 1:**
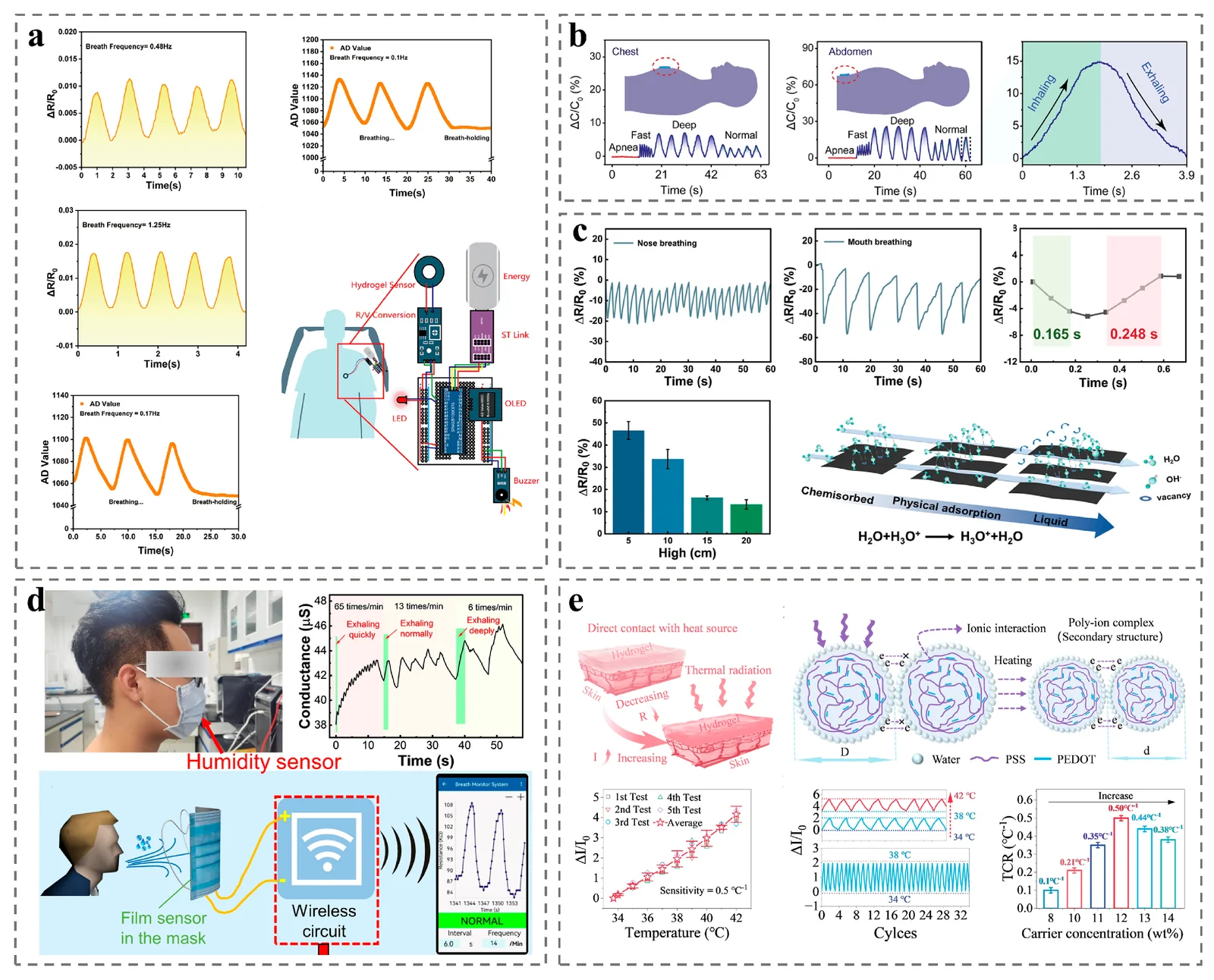
Strategies for acquiring respiratory movement and airflow signals using hydrogel-based sensors. (**a**) Monitoring of respiratory rate and status based on mechanical response. Reproduced from reference [21] under Creative Commons license CC-BY-NC-ND, copyright 2026, John Wiley and Sons. (**b**) Monitoring of respiratory rhythm and depth based on mechanical response. Reproduced with permission from reference [36], copyright 2026, John Wiley and Sons. (**c**) MXene/LiCl/PAAm/PVA organic hydrogel for monitoring respiratory airflow based on humidity response. Reproduced with permission from reference [37], copyright 2026, John Wiley and Sons. (**d**) Hydrogel film for monitoring respiratory airflow based on humidity response. Reproduced from reference [38] under Creative Commons license CC-BY, copyright 2022, Springer Nature. (**e**) Respiratory airflow detection based on temperature response. Reproduced from reference [39] under Creative Commons license CC-BY, copyright 2025, Springer Nature.

Mechanical deformations, including stretching and compression induced by thoracoabdominal movements, can be exploited to extract respiratory rate, respiratory depth, and rhythm variations, thereby enabling the identification of sleep apnea and hypopnea events [7]. By analyzing signal frequency and amplitude variations, respiratory rate and movement intensity can be quantitatively evaluated, enabling the characterization of respiratory depth and dynamic breathing patterns [36]. For instance, hydrogel-based sensors capable of real-time respiratory signal acquisition have demonstrated high consistency with conventional monitoring methods and can effectively distinguish different respiratory states, including normal breathing, apnea, and tachypnea (Figure 1a) [21]. In another study, hydrogel-based sensors evaluated thoracic and abdominal movement intensity through variations in capacitive signal amplitude. During respiration, inhalation causes the abdomen to expand and enhance the Δ*C*/*C*_0_ signal, whilst exhalation leads to a decrease in the signal. Rapid breathing generated higher-frequency signal fluctuations, whereas deep breathing resulted in larger signal amplitudes. These characteristics enable sensitive detection of respiratory rhythm and depth variations, providing effective monitoring of dynamic respiratory processes (Figure 1b) [36].

### 2.2. Respiratory Airflow Detection Based on Humidity, Thermal, and Mechanical Responses

Respiratory airflow directly reflects airway patency and ventilation status, serving as a key physiological parameter for identifying abnormal events such as obstructive sleep apnea (OSA) [5]. As respiration involves dynamic changes in water vapor exchange, temperature variation, and airflow disturbance, hydrogel-based sensors can leverage their hygroscopicity, thermal responsiveness, and mechanical performance to enable multidimensional monitoring of respiratory airflow signals [37,40,41].

Regarding humidity sensing, exhaled water vapor interacts with the hydrogel matrix, inducing water absorption and swelling of the material, which subsequently promotes ion migration and regulates electrical conductivity. These variations in electrical conductivity can be captured through resistance changes to reflect respiratory cycles [10]. Compared with mechanical sensing, this approach provides a more direct indication of respiratory airflow by exploiting the moisture difference between inhalation and exhalation [42]. However, its dependence on moisture also makes the sensing performance susceptible to environmental humidity. The changes in ambient humidity can alter the hydration state of the hydrogel and cause fluctuations in the electrical response. Moreover, the hydrogels with high water content have gradual water loss during prolonged use, resulting in changes in their mechanical and electrical properties and reduced sensing stability [37]. For instance, an MXene/LiCl/polyacrylamide (PAAm)/polyvinyl alcohol (PVA) organic hydrogel sensor enables real-time monitoring of nasal and oral respiration by detecting resistance variations induced by respiratory humidity. This sensor exhibits rapid response and demonstrates the feasibility of hydrogel-based humidity sensing for non-invasive respiratory airflow monitoring (Figure 1c) [37]. Liang et al. developed hydrogel films incorporating multiple polymer networks for humidity-based detection of nasal airflow. The sensor achieved an ultrahigh humidity sensitivity of up to 13,462.1%/%RH, surpassing previously reported hydrogel-based humidity sensors and comparable state-of-the-art humidity sensing devices. Respiratory waveforms were acquired based on conductivity variations induced by exhalation and inhalation, where exhalation increased conductivity while inhalation reduced it. The peak frequency and amplitude of the signals corresponded to respiratory rate and depth, respectively, enabling accurate discrimination of rapid breathing (65 breaths min^−1^), normal breathing (13 breaths min^−1^), and deep breathing (6 breaths min^−1^). Furthermore, after integration with a wireless circuit and Bluetooth module, the sensing system enabled real-time respiratory monitoring and automatically triggered a sleep apnea alarm when no respiratory signal was detected for longer than 10 s (Figure 1d) [38].

Temperature-based sensing relies on the temperature difference between inhalation and exhalation, where thermal variations regulate ion transport and the conductive network configuration to generate electrical responses [40]. However, the thermal response is also influenced by the surrounding thermal environment. Variations in ambient temperature can alter the temperature gradient between exhaled air and the sensor, thereby changing the amplitude of the respiratory signal and potentially affecting monitoring accuracy [43]. The composite hydrogel sensor developed by Wang et al. exploits temperature-induced enhancement of ion transport and electronic transitions, resulting in decreased resistance and increased current. It exhibits sensitive responses to respiratory temperature fluctuations while maintaining excellent cyclic stability (Figure 1e) [39].

Furthermore, the subtle pressure fluctuations and mechanical deformations generated by airflow can be converted into electrical signals through piezoresistive mechanisms. The incorporation of biomimetic micro- and nanostructures into hydrogel architectures can further enhance sensitivity toward weak airflow stimuli, enabling more accurate detection of respiratory airflow dynamics. Nanofiber hydrogel-based sensors exhibited rapid airflow response characteristics, and their response time and recovery time are 0.10 s and 0.04 s, respectively. When vertically integrated near the outlet of a nasal cannula, these sensors can simultaneously detect airflow from both nostrils, generating enhanced sensing signals and enabling rapid and sensitive discrimination of different respiratory states [41].

### 2.3. Multimodal Monitoring of Auxiliary Physiological Signals

In addition to respiratory movements and airflow, auxiliary physiological signals, including ECG, EEG, EOG, pulse signals, and sleep posture, provide valuable information for evaluating sleep states and physiological responses associated with respiratory abnormalities [7].

**Figure 2:**
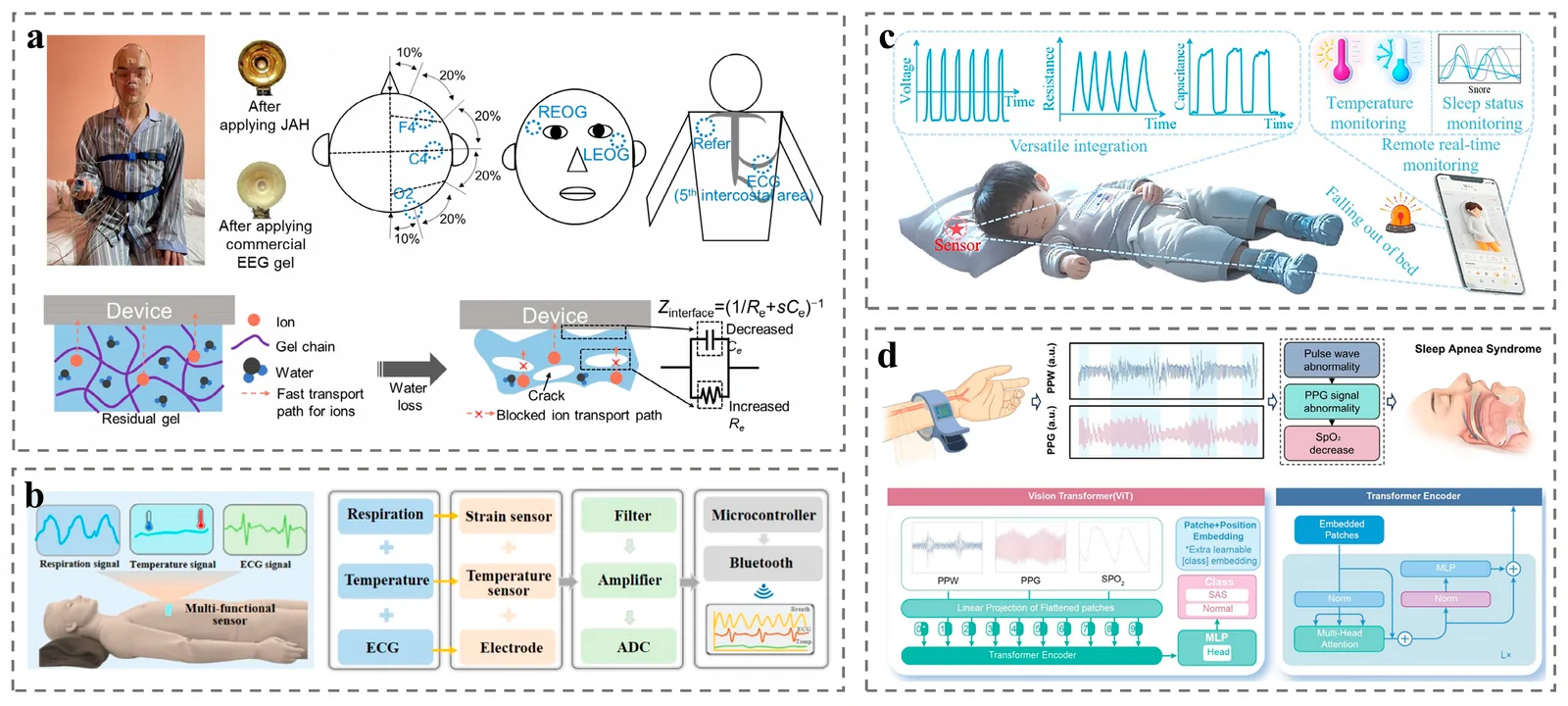
Multimodal hydrogel-based sensors for auxiliary sleep monitoring. (**a**) Hydrogel bioelectronic interfaces for clinical PSG, enabling the acquisition of electrophysiological signals, including ECG and EEG. Reproduced from reference [44] under Creative Commons license CC-BY-NC-ND, copyright 2024, Springer Nature. (**b**) Hydrogel patches for simultaneous monitoring of respiratory, temperature, and ECG signals, enabling multiparameter assessment of sleep-related physiological status. Reproduced with permission from reference [45], copyright 2026, American Chemical Society. (**c**) A multimodal hydrogel sensing system capable of temperature, pressure, and proximity sensing for monitoring sleep behaviors and physiological conditions. Reproduced from reference [39] under Creative Commons license CC-BY, copyright 2025, Springer Nature. (**d**) Schematic illustration of a dual-modal wearable system integrating pulse pressure wave, SpO_2_, and heart rate sensing with deep learning for sleep apnea classification. Reproduced with permission from reference [46] under Creative Commons license CC-BY, copyright 2025, John Wiley and Sons.

For electrophysiological signal acquisition, hydrogel-based sensors can form stable, low-impedance interfaces with the skin, enabling the reliable recording of bioelectrical signals such as ECG, EOG, and EEG [44,45]. For instance, a Janus-adhesive hydrogel bioelectronic interface enables the simultaneous acquisition of multichannel electrophysiological signals, including ECG, EOG, and EEG. Its excellent interfacial conformability and resistance to motion-induced interference ensure high-quality signal acquisition during long-term sleep monitoring. By integrating with polysomnographic analysis, this system facilitates the evaluation of sleep stages and the apnea–hypopnea index in patients with OSA, providing a reliable approach for OSA diagnosis (Figure 2a) [44]. Furthermore, a multifunctional hydrogel patch based on an island-bridge structure enables simultaneous monitoring of respiratory, temperature, and ECG signals. The mechanically decoupled island-bridge architecture minimizes motion artefacts, allowing stable acquisition of respiratory strain, electrophysiological, and temperature signals during sleep. The comprehensive analysis of these multimodal signals provides insights into ventilation status, cardiovascular regulation, and metabolic rhythms, while improving the accuracy of sleep-related breathing abnormality detection through cross-validation among different physiological parameters. This strategy offers an effective pathway toward continuous and home-based sleep health monitoring (Figure 2b) [45].

Beyond electrophysiological signals, posture and body movement information can provide additional insights into sleep behavior and contribute to the assessment of sleep-related breathing conditions [12]. A dual-layer hydrogel patch consisting of a temperature-sensing layer and a non-contact detection layer based on electrospun nanofibers can simultaneously capture pressure, proximity, and temperature signals. Combined with deep learning (DL) algorithms, this system enables recognition of different sleep states, including lying, sitting, turning over, and snoring, demonstrating the potential of multimodal hydrogel-based sensors for intelligent sleep behavior monitoring (Figure 2c) [39].

Moreover, pulse signals can reflect cardiovascular responses induced by hypoxia and autonomic nervous system regulation during sleep-related respiratory abnormalities, serving as an important complementary indicator for identifying apnea events [7,46]. For example, an alkaline-treated polyacrylonitrile/ionic liquid/electrostatic flocked-carbon fibers (aPAN/IL/CFs) hydrogel sensor can be attached to multiple body locations, including the nose, abdomen, and wrist, for simultaneous monitoring of respiratory airflow, thoracoabdominal movements, and pulse signals. The wrist-mounted sensing unit can detect subtle pulse vibrations and analyze characteristic pulse waveforms, thereby improving the identification of sleep-related breathing disorders through the integration of respiratory and cardiovascular information. In addition, the nanofiber hydrogel sensor supports gesture recognition, providing opportunities for assisted interaction and emergency communication in patients with severe sleep apnea and limited verbal communication abilities. These capabilities further expand the application prospects of multimodal hydrogel-based sensors in intelligent healthcare monitoring [41]. Based on the acquisition of multi-source physiological signals, integrating complementary pulse and blood oxygen information with deep learning-based feature extraction and state recognition can further enhance the automated identification of sleep apnea. For example, a dual-modal pulse monitoring system integrating a self-powered piezoelectric nanogenerator (PENG) and photoplethysmography (PPG) was proposed. The PENG continuously monitored pulse pressure waves and triggered the PPG module upon detecting abnormal pulse patterns to acquire blood oxygen saturation (SpO_2_) and heart rate information. A DL model was then employed to jointly analyze pulse, blood oxygen, and other physiological information, enabling automated recognition of sleep apnea states (Figure 2d) [46].

### 2.4. Signal Cross-Talk and Decoupling Strategies in Multimodal Sensing

Multimodal hydrogel-based sensors can simultaneously acquire multiple physiological signals on a single flexible platform, providing more information for the comprehensive monitoring of complex physiological states [12]. However, different physical stimuli can concurrently affect the conductive network, ion transport, and interfacial state of hydrogels, resulting in cross-responses among different sensing modalities and compromising the accurate identification of individual signals [11]. Therefore, effective multimodal sensing requires not only the simultaneous acquisition of multiple physiological signals, but also the suppression of signal crosstalk induced by different physical stimuli [47].

One direct strategy for signal decoupling is introducing spatially separated sensing regions, allowing different physical stimuli to be detected at relatively independent locations through distinct sensing mechanisms [48]. Li et al. introduced an independent hydrogel-based temperature-sensing unit adjacent to a hydrogel strain sensor. By simultaneously monitoring ambient temperature and compensating for temperature-induced variations in the strain signal, they achieved effective decoupling of temperature and strain responses. The resulting sensor exhibited a strain detection range of up to 1000% and a thermal response sensitivity of −7.16% °C^−1^ [49].

Modular and multilayered structural designs can further enhance spatial isolation between different sensing units [50,51]. For example, hydrogel sensing units with distinct functions were assembled in a modular configuration, combining polyacrylamide/chitosan strain-sensing units with polyacrylamide/sodium alginate-poly(3,4-ethylenedioxythiophene) temperature-sensing units. Ca^2+^-induced crosslinking generated differences in mechanical performance that promoted localized strain concentration, while interfacial energy filtering enhanced the temperature response, enabling independent detection of strain and temperature signals [50]. In another study, a three-layer temperature–strain sensing structure was developed. Spatial separation of the temperature-sensing layer, thermal insulation layer, and strain-sensing layer reduced heat transfer between the sensing units, while independent electrical outputs enabled effective spatial decoupling of these two signals [51].

Beyond spatial structural design, the difference in sensing mechanisms provides another effective strategy for reducing signal crosstalk. When different stimuli are transduced through distinct mechanisms, such as thermoelectric, piezoelectric, or ionic conduction, they can generate electrical outputs with different response characteristics. These distinct transduction pathways can therefore be exploited to discriminate among signals arising from different physical stimuli [52,53].

Multimodal signal decoupling should also account for the effects of dynamic mechanical noise generated by respiratory movements on the acquisition of auxiliary physiological signals [54]. Frequency-selective damping provides an effective structural modulation strategy for multimodal sleep respiratory sensing by dissipating mechanical energy within specific frequency ranges, thereby attenuating dynamic respiratory noise and minimizing its interference with auxiliary physiological signals [44].

### 2.5. AI-Assisted Signal Analysis and Sleep Respiratory State Recognition

Although multimodal hydrogel-based sensors are capable of simultaneously acquiring mechanical, humidity, temperature, and bioelectrical signals, physiological signals generated during sleep are highly complex and exhibit considerable inter-individual variability, making accurate state recognition challenging. Artificial intelligence (AI) technologies, including machine learning (ML) and deep learning (DL), provide powerful approaches for analyzing multisource signals and enabling intelligent sleep-state assessment [17,55].

ML algorithms can extract latent features from continuously acquired signals, including strain, resistance, capacitance, and temperature, and establish relationships between signal variations and different respiratory states. This capability enables the automated recognition of complex breathing patterns, such as normal breathing, deep breathing, shallow breathing, and apnea events [56]. For instance, a multimodal hydrogel sensor with dual sensitivity to strain and temperature, combined with ML algorithms, performs synergistic analysis of respiratory-induced mechanical deformation and thermal variations. By integrating feature information from multiple sensing modalities, the model effectively differentiates various respiratory patterns, achieving an average prediction accuracy of 98.4% [57].

Owing to their strong adaptability and high accuracy in processing complex datasets, DL-based methods have been increasingly applied to the intelligent interpretation of physiological signals [58]. DL algorithms can extract subtle features from respiratory signals that are difficult to be identified using conventional approaches, including frequency, amplitude, duration, waveform morphology, and temporal dynamics. These capabilities significantly improve the accuracy and real-time performance of sleep-related respiratory event recognition [59]. For example, a dual-modal hydrogel monitoring system based on respiratory and pulse signals collected data from eight volunteers and employed a gated recurrent neural network to classify OSA-hypopnea syndrome. The fusion model based on respiratory-pulse bimodal information achieved training, testing, and validation accuracies of 85.6%, 85.6%, and 77.8%, respectively. Considering the high temporal dependence between respiratory and pulse signals, a bidirectional gated recurrent neural network was further introduced to exploit bidirectional temporal features, improving the training, testing, and validation accuracies to 100%, 99.4%, and 98.9%, respectively, while achieving rapid recognition within 22.3 ms [17].

## 3. Structural Design Strategies for Hydrogel-Based Multimodal Sleep Respiratory Monitoring

Multimodal sleep respiratory monitoring requires the simultaneous acquisition of mechanical movements, respiratory airflow, and diverse physiological signals, thereby imposing higher requirements on the structural design of hydrogel-based sensors [12,36]. Through precise regulation of hydrogel network architectures, functional layer integration, and device assembly strategies, researchers have developed various structural approaches, including single-layer, multilayer, biomimetic, and integrated patch designs. These strategies aim to enhance sensing sensitivity, signal selectivity, and long-term wearing comfort, providing a critical foundation for the development of high-performance flexible sleep monitoring systems [41,45,60]. Table 1 summarizes the structural features, key performance metrics, and representative applications of hydrogel-based sensors with different architectures for sleep respiratory monitoring, offering insights into the relationship between structural design and sensing performance.

### 3.1. Single-Layer Structures

Single-layer hydrogels primarily utilize internal ionic or electrically conductive networks to convert mechanical stimuli into electrical signals. Meanwhile, the abundant hydrophilic functional groups and ionic migration characteristics within hydrogel matrices enable responses to respiratory-induced humidity and temperature variations, facilitating respiratory airflow monitoring [12,37]. For example, a polyvinyl alcohol/sodium alginate/starch-based ionically conductive hydrogel constructs a flexible single-layer network through hydrogen bonding and ionic cross-linking, and is integrated with a supercapacitor structure to achieve dual-mode resistive–capacitive signal output. The resistive signal reflects deformation-induced variations in the conductive network, enabling the detection of respiratory movements, whereas the capacitive signal responds to temperature fluctuations associated with respiratory airflow through temperature-dependent ion migration and interfacial polarization changes, allowing nasal airflow monitoring. Furthermore, by incorporating artificial neural network algorithms, the system extracted characteristic features from complex voltage signals and achieved high-precision recognition of gestures and postures, with an accuracy of 99.259% [12].

**Figure 3:**
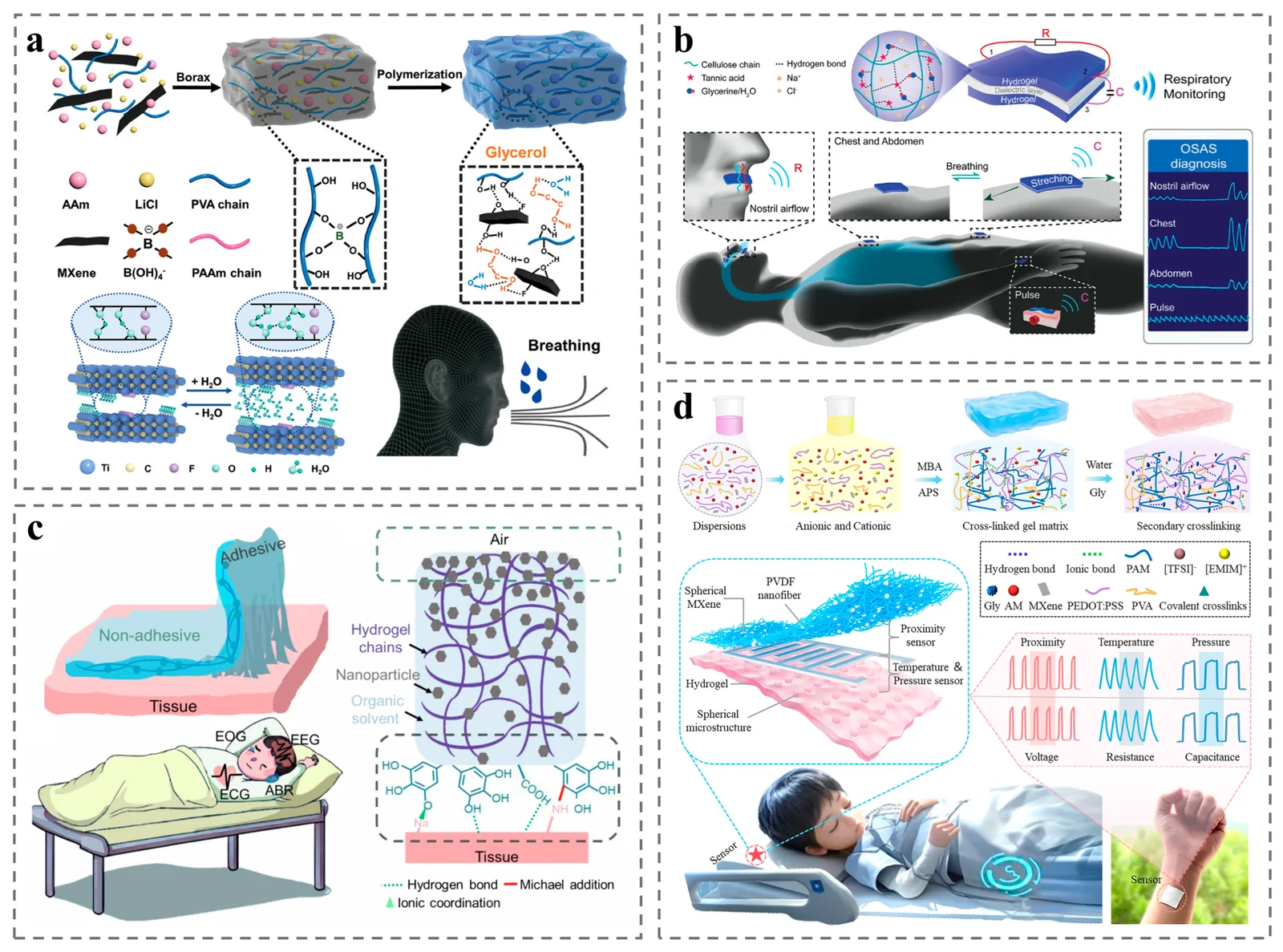
Structural design strategies of multimodal hydrogel-based sensors for sleep-breathing monitoring. (**a**) A single-layer MXene/LiCl/PAAm/PVA hydrogel enabling simultaneous detection of humidity and mechanical signals. Reproduced with permission from reference [37], copyright 2024, John Wiley and Sons. (**b**) A CH-GT sandwich-structured hydrogel enabling dual-mode monitoring of respiratory movements and airflow. Reproduced with permission from reference [36], copyright 2026, John Wiley and Sons. (**c**) A Janus-adhesive hydrogel interface enabling multichannel acquisition of electrophysiological signals. Reproduced from reference [44] under Creative Commons license CC-BY-NC-ND, copyright 2024, Springer Nature. (**d**) A bilayer integrated hydrogel patch enabling the fusion of pressure, temperature, and motion signals. Reproduced from reference [39] under Creative Commons license CC-BY, copyright 2025, Springer Nature.

The multimodal sensing capability of single-layer hydrogels can be further enhanced through the incorporation of functional materials and microstructural engineering. For instance, the MXene/LiCl/PAAm/PVA hydrogel employs MXene to establish a conductive network and LiCl to promote ionic migration, enabling simultaneous detection of humidity and mechanical signals for monitoring respiratory airflow and movements (Figure 3a) [37]. In another study, an open macroporous deep eutectic solvent hydrogel developed through a “lock–unlock” strategy dynamically regulates its network structure to improve gas transport and interfacial responsiveness, enabling trimodal sensing of humidity, temperature, and strain. Specifically, metal ions first coordinate with hydroxyl groups on PVA chains to construct a stable “locked” network, enhancing the hydrogel’s structural integrity. Subsequently, citrate ions interact with metal ions to weaken the cross-linking constraints and induce salt precipitation, generating a uniform open-pore structure. This architecture facilitates the simultaneous acquisition of humidity, temperature, and mechanical strain signals. As a result, the single-layer hydrogel enables coordinated monitoring of airflow variations, respiratory rhythms, and body movements during sleep breathing without requiring complex functional separation or single-layer integration [10].

Single-layer hydrogel architectures simplify device fabrication while maintaining multimodal sensing capabilities, providing an important foundation for lightweight and flexible sleep monitoring systems. However, because multiple external stimuli are often applied to the same sensing network, single-layer designs still face challenges associated with signal interference and accurate decoupling of multiple sensing parameters [45].

### 3.2. Multilayer Structures

Multilayer hydrogel architectures enable the independent acquisition of multiple physiological signals through the spatial separation of functional layers. Compared with single-layer designs, multilayer architectures allow the sensing layer, dielectric layer, and electrode components to be independently optimized according to the characteristics of different target signals, thereby reducing signal interference and improving long-term monitoring performance during sleep [36,39].

The key advantages of multilayer architectures lie in functional layer separation and effective signal decoupling [63]. For example, a dual-mode sensor based on cellulose hydrogel employs a sandwich architecture consisting of two CH-GT hydrogel layers, an intermediate dielectric layer, and integrated electrodes, forming a capacitive-resistive dual-mode sensing system. The capacitive sensing layer primarily responds to mechanical stimuli, including thoracoabdominal movements and pulse signals, whereas the resistive sensing layer detects temperature variations associated with nasal airflow. Through independent signal outputs from different sensing channels, this architecture effectively distinguishes mechanical and thermal responses and enables real-time monitoring of OSAS (Figure 3b) [36]. The combination of multilayer structural design and adhesive interfaces further enhances the capability of hydrogel-based sensors to capture complex physiological information during sleep [64]. Luo et al. developed a Janus-adhesive hydrogel with selective damping performance by introducing a gradient distribution of silver nanoparticles. This asymmetric structure was achieved through the gravitational sedimentation of silver nanoparticles during gelation, inhibition of radical coordination, and subsequent resedimentation induced by the Trommsdorff–Norrish effect. The low-silver-content region retains abundant catechol groups, enabling strong adhesion to the skin through Michael addition, hydrogen bonding, and ionic coordination. In contrast, the high-silver-content region enhances energy dissipation through reversible weak interactions between silver nanoparticles and polymer chains. This gradient architecture selectively attenuates dynamic noise within the respiratory frequency range (0.1–1 Hz), reduces motion artefacts and interfacial impedance, and improves the reliability of long-term bioelectrical signal acquisition, including EEG and ECG. Consequently, it provides a promising approach for sleep-stage analysis and auxiliary diagnosis of sleep apnea (Figure 3c) [44].

Multilayer architectures can further broaden sleep behavior monitoring capabilities through the integration of diverse functional materials. For instance, a flexible integrated multimodal pressure-temperature sensing patch adopts a bilayer configuration that functionally separates a hydrogel-based pressure-temperature sensing layer from an electrospun nanofiber-based non-contact detection layer. The hydrogel layer has enhanced local stress sensitivity through microsphere structures and incorporates PEDOT: PSS for temperature sensing, while the upper nanofiber layer employs MXene-doped triboelectric structures and interdigital electrodes for non-contact motion detection. This multilayer design effectively minimizes interference between sensing modalities, enabling simultaneous acquisition of pressure, temperature, and motion signals. Combined with DL algorithms, the system facilitates the recognition of sleep postures and abnormal sleep behaviors (Figure 3d) [39].

Multilayer hydrogel architectures offer significant advantages in improving signal accuracy and reducing cross-interference among sensing modalities. However, challenges remain, including complex fabrication processes and long-term interfacial stability between different layers [45]. Future advances in interlayer bonding strategies and intelligent signal-processing algorithms are expected to further enhance the performance of multilayer hydrogel sensing systems for high-precision and long-term sleep apnea monitoring [17].

### 3.3. Biomimetic Structures

By mimicking the structural characteristics of natural sensory systems, such as human skin and hair, biomimetic architectures enhance the ability of hydrogel-based sensors to detect subtle physiological stimuli, providing new design strategies for highly sensitive and multiparameter sleep monitoring [41,65]. Compared with conventional planar designs, biomimetic architectures employ micro- and nanostructures to amplify local deformation and interfacial variations induced by external stimuli, thereby improving sensitivity toward weak airflow, mechanical vibrations, and physiological signals [66].

**Figure 4:**
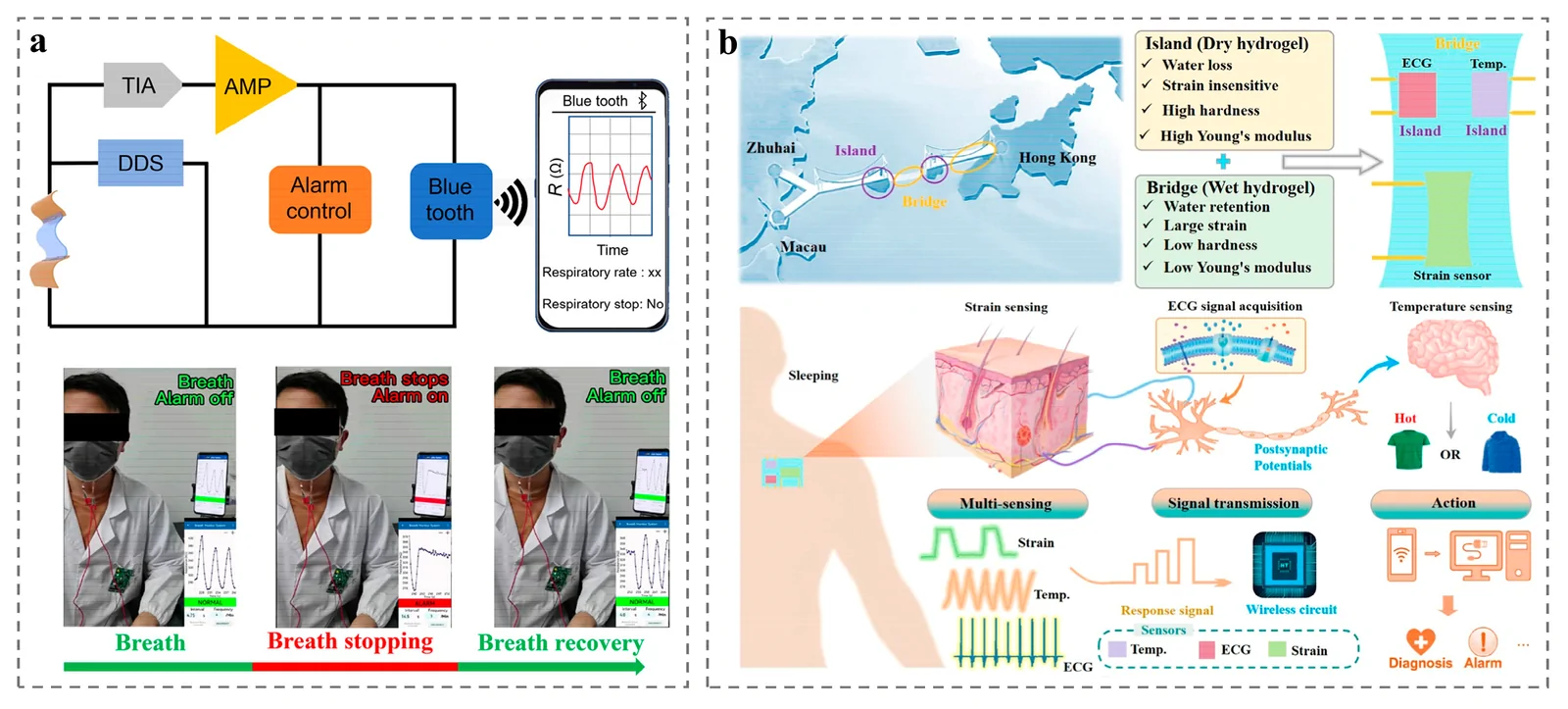
Applications of biomimetic and integrated hydrogel sensor architectures in sleep apnea monitoring. (**a**) Biomimetic ionic electro-hydrogels exhibit excellent humidity-sensing performance, making them promising for wireless respiratory monitoring. Reproduced with permission from reference [61] under Creative Commons license CC-BY, copyright 2022, John Wiley and Sons. (**b**) An integrated hydrogel patch with an island-bridge architecture that employs functional zoning to acquire temperature, ECG, and respiratory signals while enhancing resistance to motion-induced interference. Reproduced with permission from reference [45], copyright 2026, American Chemical Society.

For example, the aPAN/IL/CFs nanofiber hydrogel sensor achieved skin- and hair-inspired sensing by integrating an aPAN/IL nanofiber hydrogel substrate with vertically aligned carbon fiber arrays. The aPAN/IL hydrogel mimics the function of skin and neural networks by providing continuous conductive pathways and strong biointerfacial adhesion, whereas the electrostatically flocked carbon fiber array reproduces the hair-like sensing structure. The changes in contact and separation between individual fibers amplify responses to weak airflow and mechanical strain. This biomimetic architecture enables multimodal detection of oral and nasal respiratory airflow, abdominal respiratory movements, and sleep pulse signals. It exhibits rapid airflow response (0.10 s) and high strain sensitivity (gauge factor, GF = 76.1), making it suitable for real-time sleep apnea monitoring [41]. In addition, biomimetic design can enhance humidity sensing by constructing ion-sensitive interfaces that mimic the functional characteristics of human skin. For example, a biomimetic ion skin based on a PVA–cellulose nanofiber (CNF) organic hydrogel film promotes ion migration and interfacial charge transfer upon water molecule adsorption through microstructural regulation, thereby enabling a highly sensitive, rapid, and stable humidity response. The sensor enables real-time respiratory monitoring and can be further integrated with wireless circuits to construct a sleep respiratory monitoring system. An alarm is triggered after 10 s of continuous apnea, demonstrating the potential of biomimetic hydrogel-based sensors for wireless sleep respiratory monitoring (Figure 4a) [61].

Biomimetic architectures have enhanced capability of hydrogel-based sensors to amplify, transmit, and resolve weak physiological signals. However, challenges in fabrication complexity, structural uniformity, and large-scale integration remain [67]. Further optimization of scalable manufacturing strategies and structural reproducibility will be essential for their future practical applications in wearable sleep monitoring systems [68].

### 3.4. Integrated Structures

Integrated architectures enable the simultaneous acquisition of multisource physiological information by incorporating multiple sensing functions into a single flexible device, representing an important strategy for improving the overall performance of sleep apnea monitoring systems [45]. Through rational structural design, integrated architectures can minimize interference among different sensing modules and enhance monitoring reliability during long-term wearable applications [69].

Liu et al. developed a multifunctional hydrogel sensor patch based on an island-bridge architecture, achieving zoned acquisition of multimodal signals through the construction of functional regions with distinct mechanical performance. The sensor employed a single polyacrylic hydrogel matrix to form low-water-content “island” regions and high-water-content “bridge” regions. The island regions integrate temperature sensors and ECG electrodes, where increased stiffness suppresses strain-induced interference and enables stable acquisition of temperature and electrophysiological signals. In contrast, the bridge regions maintain high flexibility and water retention, functioning as highly sensitive strain-sensing units for respiratory movement monitoring. By tuning the ratio of glycerol/ethylene glycol, the mechanical performance of different regions can be selectively regulated, enabling effective signal decoupling without the requirement for complex computational algorithms. This integrated architecture allows simultaneous monitoring of body temperature, ECG, and respiratory signals while maintaining low noise and high signal stability during physical activity, providing a reliable strategy for long-term home-based OSA monitoring (Figure 4b) [45].

Through regulation of functional zoning and mechanical performance, integrated hydrogel architectures achieve synergistic acquisition of multiparameter physiological signals while effectively addressing signal coupling and motion artefacts commonly encountered in conventional multifunctional sensors [50,62].

## 4. Conclusions and Outlook

The structural design of hydrogel-based sensors plays a critical role in achieving high-performance sleep apnea monitoring. In recent years, researchers have achieved synergistic optimization of hydrogel and device performance through single-layer, multilayer, biomimetic, and integrated architectural strategies. For single-layer architectures, the material’s composition and internal conductive networks are regulated to enable multimodal sensing of mechanical, humidity, and temperature signals while maintaining simple device configurations. For multilayer architectures, interference among different sensing modalities is minimized through functional layer separation, thereby improving the accuracy of multiparameter signal acquisition. Biomimetic architectures, inspired by sensory systems, such as skin and hair, employ micro- and nanostructures to obtain enhanced responses to weak respiratory airflow and mechanical stimuli. Integrated architectures further combine functional zoning, flexible interfaces, and intelligent algorithms to achieve comprehensive monitoring of respiratory dynamics, electrophysiological signals, and environmental parameters. Collectively, these structural strategies have driven the evolution of hydrogel-based sensors from single-signal detection platforms toward multimodal, interference-resistant, and long-term wearable monitoring systems.

Although structural engineering has significantly improved the monitoring capabilities of hydrogel-based sensors, their suitable applications in complex sleep environments remain challenging. From a clinical perspective, systematic validation of multimodal hydrogel-based sensors against standard PSG remains limited. Although some studies have demonstrated human sleep monitoring, respiratory event recognition, and AHI evaluation, the available evidence remains limited in terms of participants, monitoring duration, and direct or synchronous comparisons with standard PSG. Further clinical studies are therefore required to more comprehensively assess the clinical reliability and diagnostic potential of these sensors for sleep respiratory monitoring. From a practical perspective, dehydration and swelling of hydrogels may alter their mechanical and electrical performance, and contribute to long-term signal drift. Repeated deformation during prolonged monitoring may also lead to mechanical fatigue, while insufficient or excessive skin adhesion can compromise signal stability, wearing comfort, and skin compatibility. In addition, long-term biocompatibility and device-to-device reproducibility are important considerations for practical applications.

Future research should include more participants and focus on longer-term or overnight continuous sleep monitoring, additionally, synchronous measurements with standard PSG should be strengthened to further evaluate the clinical reliability and diagnostic potential of multimodal hydrogel-based sensors. Meanwhile, further efforts should focus on improving water retention, resistance to swelling, mechanical durability, skin adhesion, long-term signal stability, biocompatibility, and fabrication reproducibility. In the future, the development of hydrogel sensors with enhanced selectivity, stability, and adaptability, integrating biomimetic microstructures, intelligent device architectures, and AI-based signal analysis will help advance these systems from laboratory-based performance validation and human feasibility studies, toward more rigorous clinical validation and practical applications in continuous sleep respiratory monitoring and personalized health management.

## Figures and Tables

**Table 1:** Structural design strategies and sensing characteristics of multimodal hydrogel-based sensors for sleep respiratory monitoring.

Structures	Structural Features	Sensing Mechanisms	Sensing Modality	Detection Range	Cyclic Stability	Sensitivity	Response Time	Recovery Time	Applications	References
Single-layer structures	“Egg-box” structure	Piezoresistive	Tensile strain	0–400%	1000 cycles (100%)	NR	NR	NR	Respiratory movements, airflow temperature, and posture	[12]
		Piezoresistive	Compressive strain	0–80%	1000 cycles (50%)	NR	NR	NR		
		Resistive temperature sensing	Temperature	16–45 °C	NR	−0.084% °C^−1^	NR	NR		
		Capacitive	Tempera-ture, strain	NR	1000 cycles (5 mA cm^−2^)	NR	NR	NR		
Single-layer structures	Double network structure	Resistive humidity sensing	Humidity	40–85% RH	10,500 s (40–70% RH)	−103.4%/% RH	0.165 s	0.248 s	Airflow humidity, respiratory movements	[37]
		Piezoresis-tive, capacitive	Strain	NR	NR	NR	NR	NR		
Single-layer structures	Porous structure	Resistive humidity sensing	Humidity	11–98% RH	NR	GF = 1.45	Breathe out: 0.48 s; Breathe in: 0.4 s	NR	Respiratory movements, airflow temperature, and humidity	[10]
		Resistive temperature sensing	Temperature	0–80 °C	NR	GF = 3.39				
		Piezoresistive	Strain	0–100%	NR	GF = 3.22				
Multilayer structures	Microspherical structure, fork-finger-like electrode layer	Resistive temperature sensing	Temperature	34–42 °C	32 cycles	0.5 °C^−1^	NR	17 s (38–42 °C)	Posture, body temperature, pressure, and proximity	[39]
		Capacitive	Pressure	25–7000 Pa	16,500 cycles	30.6 kPa^−1^ (1 kPa), 26.3 kPa^−1^ (40 kPa)	<0.0056 s	<0.0056 s		
		Friction	Proximity	2 m	4000 cycles	NR	NR	NR		
Multilayer structures	Dual-network sandwich structure	Resistive temperature sensing	Temperature	20–100 °C	6400 s (2.5 kPa, 60 °C/−40 °C)	−0.83% °C^−1^	NR	NR	Respiratory movements, airflow temperature	[36]
		Capacitive	Compression	0–5 kPa		0.053 kPa^−1^ (0–45 kPa), 0.01 kPa^−1^ (45–300 kPa)	0.02 s (0.7 kPa)	NR		
Multilayer structures	Selective frequency damping, asymmetric adhesion	Electrophysiological sensing	Electrophysiological sensing	NR	10,000 cycles	NR	NR	NR	ECG, EOG, EEG, and auditory brainstem response	[44]
Biomimetic structures	Skin- and hair-inspired nanofiber structure	Piezoresistive	Airflow velocity	0.033–5.0 m s^−1^	400 cycles (0.199 m s^−1^)	38.0% s m^−1^ (ν < 1.0 m s^−1^), and 18.2% s m^−1^ (ν ≥ 1.0 m s^−1^)	0.1 s	0.04 s	Airflow, respiratory movements, and pulse	[41]
			Tensile strain	0–20%	250 cycles (2%)	GF = 20.1 (0–7%), GF = 36.9 (7–13%), GF = 76.1 (13–20%)	0.6 s	0.8 s		
Biomimetic structures	Skin-biomimetic-sensing iontronics	Capacitive	Humidity	22–98% RH	120 days	25,000% (98% RH)	380 s	140 s	Respiratory movements and humidity	[61]
		Piezoresis-tive	Strain	10–100%	NR	GF = 3.141	NR	NR		
Integrated structures	Island-bridge structure	Piezoresistive	Strain	0–180%	1000 cycles (0–50%)	GF = 5.12 (0–50%), and GF = 23.31 (60–180%)	0.198 s	0.26 s	Respiratory rate, body temperature, EMG, and ECG	[45]
		Resistive temperature sensing	Temperature	25–45 °C	1000 cycles (25–40 °C)	4.02 °C^−1^	1.25 s	NR		
		Electrophysiological sensing	Electrophysiological sensing	NR	NR	NR	NR	NR		
Integrated structures	Integrated mask	Chemiresistive	CO_2_	100–10,000 ppm	NR	NR	73.08 s	79.98 s	Respiratory CO_2_, movements, temperature, and humidity	[62]
		Resistive humidity sensing	Humidity	23–75% RH	NR	NR	NR	NR		
		Piezoresistive	Strain	0–50%	3000 cycles (30%)	GF = 0.81	NR	NR		

GF, gauge factor; RH, relative humidity; NR, not reported.

## Data Availability

No new data were created or analyzed in this study. Data sharing is not applicable to this article.

## Abbreviations

The following abbreviations are used in this manuscript:

OSAS	Obstructive sleep apnea syndrome
PSG	Polysomnography
EEG	Electroencephalograms
EOG	Electrooculograms
EMG	Electromyography
ECG	Electrocardiograms
AHI	Apnea–hypopnea index
OSA	Obstructive sleep apnea
PAAm	Polyacrylamide
PVA	Polyvinyl alcohol
DL	Deep learning
aPAN/IL/CFs	Alkaline-treated polyacrylonitrile/ionic liquid/electrostatic flocked-carbon fibers
PENG	Piezoelectric nanogenerator
PPG	Photoplethysmography
AI	Artificial intelligence
ML	Machine learning
GF	Gauge factor
CNF	Cellulose nanofiber
RH	Relative humidity
NR	Not reported
